# Integrated analysis of necroptosis-related genes for evaluating immune infiltration and colon cancer prognosis

**DOI:** 10.3389/fimmu.2022.1085038

**Published:** 2022-12-22

**Authors:** Wei Yang, Shuaibing Lu, Liangqun Peng, Zhandong Zhang, Yonglei Zhang, Dandan Guo, Fei Ma, Yawei Hua, Xiaobing Chen

**Affiliations:** ^1^ Department of General Surgery, The Affiliated Cancer Hospital of Zhengzhou University, Henan Cancer Hospital, Zhengzhou, China; ^2^ Department of Radiology, The Third Affiliated Hospital of Zhengzhou University, Zhengzhou, China; ^3^ Department of Medical Oncology, The Affiliated Cancer Hospital of Zhengzhou University, Henan Cancer Hospital, Zhengzhou, China; ^4^ Zhengzhou Key Laboratory for Precision Therapy of Gastrointestinal Cancer, Zhengzhou, China

**Keywords:** weighted gene co-expression network analysis (WGCNA), tumor microenvironment, tumor immune infiltrating cells, copy number variation (CNV), nomogram, calibration curves, ceRNA networks

## Abstract

**Background:**

Colon cancer (CC) is the second most common gastrointestinal malignancy. About one in five patients have already developed distant metastases at the time of initial diagnosis, and up to half of patients develop distant metastases from initial local disease, which leads to a poor prognosis for CC patients. Necroptosis plays a key role in promoting tumor growth in different tumors. The purpose of this study was to construct a prognostic model composed of necroptosis-related genes (NRGs) in CC.

**Methods:**

The Cancer Genome Atlas was used to obtain information on clinical features and gene expression. Gene expression differential analysis, weighted gene co-expression network analysis, univariate Cox regression analysis and the least absolute shrinkage and selection operator regression algorithm were utilized to identify prognostic NRGs. Thereafter, a risk scoring model was established based on the NRGs. Biological processes and pathways were identified by gene ontology and gene set enrichment analysis (GSEA). Further, protein-protein interaction and ceRNA networks were constructed based on mRNA-miRNA-lncRNA. Finally, the effect of necroptosis related risk score on different degrees of immune cell infiltration was evaluated.

**Results:**

CALB1, CHST13, and SLC4A4 were identified as NRGs of prognostic significance and were used to establish a risk scoring model. The time-dependent receiver operating characteristic curve analysis revealed that the model could well predict the 1-, 3-, and 5-year overall survival (OS). Further, GSEA suggested that the NRGs may participate in biological processes, such as the WNT pathway and JAK-Stat pathway. Eight key hub genes were identified, and a ceRNA regulatory network, which comprised 1 lncRNA, 5 miRNAs and 3 mRNAs, was constructed. Immune infiltration analysis revealed that the low-risk group had significantly higher immune-related scores than the high-risk group. A nomogram of the model was constructed based on the risk score, necroptosis, and the clinicopathological features (age and TNM stage). The calibration curves implied that the model was effective at predicting the 1-, 3-, and 5-year OS of CC.

**Conclusion:**

Our NRG-based prognostic model can assist in the evaluation of CC prognosis and the identification of therapeutic targets for CC.

## 1 Introduction

Colon cancer (CC) is a deadly tumor that affects individuals worldwide. The incidence of CC is increasing, especially in cities and regions with rapid economic development in the United States (1). With the popularization of cancer screening and advancements in treatment-related medical technology, patient outcomes have improved significantly. But as the onset of CC is insidious, there’s still a lot patients diagnosed in the advanced stage, where the condition is severe and difficult to treat, and palliative care is the only available treatment option (2, 3). Only few biomarkers are available for the diagnosis and therapy of CC. Circulating tumor DNA (ctDNA) is a subset of circulating free DNA (cfDNA) from tumor cells. In many studies, ctDNA has been found to be of great value in the early diagnosis, efficacy evaluation, drug resistance monitoring and prognosis prediction of tumors. Among them, targeted drug therapy guided by ctDNA is the most important clinical application at present (4, 5). At present, ctDNA as biological markers have been found to be associated with the prognosis of colon cancer, but they have not been widely applied in clinic (6–8). Therefore, exploring potential biomarkers of CC remains the focus of CC-related research.

Necroptosis is a lytic manner of programmed cell death that prevents the self-destruction of activated cells that are blocked by apoptosis. In some degenerative or inflammatory diseases, necrotizing apoptosis plays a role in destroying infected cells or damaged cells (9). Unlike apoptosis, the activation of necroptosis does not depend on caspase kinase activation. Under caspase inhibition, the binding of death receptor and ligand can trigger necrotizing apoptosis (10). Necrotizing apoptosis plays a dual role in tumorigenesis and development, which can not only enhance cellular immunity (11) and play an anti-tumor role, but also stimulate the tumor to form an immunosuppressive microenvironment and promote tumor progression (12). According to previous studies (13) and owing to the activity of intracellular RIP-1 and MLKL, the combination of 5-FU and ZVAD (caspase inhibitor) can promote necroptosis of colorectal cancer (CRC) cells, highlighting the important value of necroptosis in the study of tumor drug resistance. However, a prognostic scoring system for CC based on the tags of genes associated with necroptosis has not been established.

Recently, high-throughput sequencing and the gene chip technology have been widely used in the field of life science (14, 15). Bioinformatics is an important tool for analyzing large volumes of existing biological data. By analyzing the potentially important core genes or prognostic factors within the data (16, 17), potential tumor markers or therapeutic targets can be explored (18). Several previous studies focused on single genes as diagnostic and prognostic indicators (19, 20). However, these biomarkers, especially individual gene expression levels that may be influenced by multiple factors, are insufficient to accurately and independently predict patient outcomes. As a result, these markers cannot be used as reliable and independent prognostic indicators. Therefore, in this study, statistical models composed of multiple prognostic necroptosis-related markers were employed to improve the predictive power of CC.

In recent years, the ceRNA hypothesis has attracted attention and has become one of the hot spots in the study of RNA interaction. The regulatory mechanisms among mRNA, miRNA, lncRNA, or circRNA are extremely complex and have important biological significance. LncRNA or circRNA can compete with mRNA to bind to miRNA, thereby forming a complex lncRNA-miRNA-mRNA network or circRNA-miRNA-mRNA network. However, an imbalance in the ceRNA regulatory network can lead to the initiation and progression of tumors (21). To date, the function of most mRNAs as ceRNAs in the progression and prognosis of CC has not been thoroughly defined.

In this study, the prognostic risk model of necroptosis-related genes (NRGs) was established, and the diagnostic and predictive significance of the model was evaluated. Thereafter, a ceRNA network was constructed based on mRNA-miRNA-lncRNA, and the effects of necroptosis-related risk score on different degrees of immune cell infiltration were evaluated. Overall, the findings of this study provide a theoretical foundation for further assessments of the diagnosis, treatment, and molecular mechanism of CC.

## 2 Materials and methods

### 2.1 Data download

The Cancer Genome Atlas (TCGA) Genomic Data Commons (GDC) website (https://portal.gdc.cancer.gov/) was used to obtain the expression spectrum data for colon cancer (colon adenocarcinoma, COAD) patients (n = 514), such as the count, and FPKM and TPM values; and patient clinical data (n=430), such as gender, age, TMN stage, and survival prognosis. “Masked somatic mutation” was selected as the somatic mutation data (n=420) and downloaded. The somatic mutations were visualized using maftools package (22) in R to obtain tumor mutation burden (TMB) for per patient. Additionally, the MSI data in the TCGA-COAD patients’ dataset was obtain the tumor mutation burden (TMB) per patient. Additionally, the MSI data in TCGA-COAD patient dataset were obtained from the cBioPortal database (https://www.cbioportal.org). The baseline information of TCGA-COAD patients is provided in **Table 1**.

**Table 1:** Baseline data table of patients in TCGA-COAD dataset.

Characteristic	levels	Overall
n		363
status, n (%)	Alive	279 (80.9%)
	death	66 (19.1%)
Age, n (%)	<60	96 (26.4%)
	**≥**60	267 (73.6%)
Gender, n (%)	female	177 (48.8%)
	male	186 (51.2%)
Stage, n (%)	stage I	62 (17.4%)
	stage II	145 (40.7%)
	stage III	103 (28.9%)
	stage IV	46 (12.9%)

Gene expression data and the clinical characteristics of patients of GSE17536 (23) and GSE39582 (24) were downloaded from the GEO database. The data samples were obtained from *Homo Sapiens*. The chip platforms were grounded in the GPL570 [HG-U133_plus_2] Affymetrix Human Genome U133 Plus 2.0 Array. After deleting patients lacking clinical information, 177 COAD tissue samples were included in GSE17536, all of which were used in the analysis. GSE39582 included 585 COAD tissue samples. As survival information was not available for 5 samples, 580 were included in the analysis. R’s limma package (25) was used to standardize the two data sets separately. **Table S1** shows the information from GEO.

### 2.2 Calculation of the necroptosis score based on gene expression matrix

For all samples in the combined dataset and based on 36 necrotizing apoptosis-related genes from previously published literature (26), the necroptosis score (NPs) of every sample in the TCGA-COAD dataset was determined using the R package, GSVA (27), and the ssGSEA method according to the gene expression matrix of the respective sample.

### 2.3 Screening of differentially expressed genes (DEGs)

Using the above method, the NPs of each sample was obtained, and the optimal cut-off value was selected in combination with the patient’s survival data. Based on the NPs score, the NPs group was separated into high and low NPs. To identify genes associated with NPs, the DEGs between high NPs and low NPs in TCGA-COAD samples were identified using the R package, limma (25). The screening threshold of the DEGs was set to |log2 fold change (FC)|>1 and adjusted P < 0.05. The results of the difference analysis are presented as a heatmap and a volcano plot.

### 2.4 Weighted gene co-expression network analysis (WGCNA)

WGCNA was implemented using the R package, WGCNA (28). First, the weighted values of the calculated correlation coefficients between any two genes were used to generate connections between genes in the network to assemble a scale-free network. A hierarchical clustering tree was then established according to the correlation coefficients. The branches of the cluster tree highlighted various genetic modules, and various colors signified different modules. The module saliency was then calculated. All mRNAs of the sample were input into WGCNA to measure the associations between the two NPs groups and different modules. All genes were recorded in their respective modules. The genes in the respective modules were considered as modular characteristic genes (MEs). The correlation between the NPs values and genes was determined based on the significance of genes. Module membership was determined according to the relevance between module genes and DEG expression profile. The modules of interest were selected using the MS score, and all genes in these modules were recognized to have a high correlation with NPs.

### 2.5 Subtype analysis of patients with CC based on necroptotic genes

Based on the necroptosis characteristic genes and TCGA-COAD expression data, the k-means method in “ConsensusClusterPlus” R package (29) was used to perform unsupervised cluster analysis to identify the necroptosis subtypes. The concordant clustering algorithm was used to discern the cluster number. The analysis was repeated 1000 times to ensure stability of the category. Principal component analysis (PCA) was conducted for patients with grouped subtypes to determine the differences between samples. Survival analysis was implemented after grouping to determine the influence of various subtypes on prognosis.

### 2.6 Establishment and testing of the prognostic risk models based on NPs characteristic genes

The results of differential expression analysis combined with WGCNA analysis were used to acquire the NPs-related characteristic genes. The significantly differentially expressed NPs-related characteristic genes were involved in the model, and the prognostic genes were screened using univariate Cox regression analysis, with a cut-off P value of 0.1. Subsequently, the selected genes were regularized and dimensionally reduced using the least absolute shrinkage and selection operator regression (LASSO) algorithm to further identify prognostic-related genes. Thereafter, the weighted normalized gene expression value of the penalty coefficient acquired by multivariate Cox analysis (STEP method) was used to establish a risk score formula. Using the median risk score, patients were divided into high-risk and low-risk groups.


$$\text{risk}Score=\sum_{i}Coefficient\;(hub\;gene_{i})*mRNA\;Expression\;(hub\;gene_{i})$$

The above dataset based on TCGA-COAD served as a training set, and the internal test was conducted using the bootstrap method with 1000 re-sampling. Thereafter, the coefficient based on model variables was used to calculate the risk score for each sample in test sets, GSE17536 and GSE39582, using the predict function in the “survival” R package (https://CRAN.R-project.org/package=survival>). Finally, a time-dependent receiver operating characteristic (ROC) curve was plotted. The area under the curve (AUC) was used to reflect the performance of the model.

### 2.7 Analysis of DEGs in the NPs-related metabolic model

To acquire genes relevant to the NPs model, DEGs between the high-risk group and low-risk group of TCGA-COAD patients were analyzed using the R limma package. The screening threshold of the significantly different DEGs was defined as |LogFC| > 1 and adj. P value < 0.05. The DEGs were visualized using volcano maps and heat maps.

### 2.8 Functional enrichment analysis

GO (30) analysis is an approach adopted for massive functional enrichment research, including biological processes (BP), molecular functions (MF), and cellular components (CC). Kyoto Encyclopedia of Genes and Genomes (KEGG) (31) is an extensively used database that stores data regarding genomes, biological pathways, diseases, and drugs. The ClusterProfiler package of R (32) was used for GO and KEGG analyses of significant DEGs. The critical value of FDR less than 0.05 indicated significant difference.

To explore the discrepancies in biological processes among different subgroups, GSEA was performed according to the gene expression profiling dataset of COAD patients. The gene set “c2.cp.v7.2.Symbols. gmt” obtained from the MSigDB (33) database was used for GSEA. An FDR < 0.25 indicated statistical significance.

### 2.9 Identification and correlation analysis of the tumor immune infiltrating cells

To quantitatively analyze the relative tumor infiltration degree of various immunocytes in COAD, the ssGSEA algorithm was employed to differentiate between highly sensitive and specific phenotypes of various human immune cells in the tumor microenvironment (TME). The algorithm revealed 28 gene sets for labeling various tumor-infiltrating immunocyte types based on a study by Bindea et al. (34). The gene sets comprised various human immunocyte subtypes, such as macrophages, mast cells, etc. Enrichment scores obtained using ssGSEA in R’s GSVA package indicated the degree of infiltration of various immune cell types in every sample. Meanwhile, R’s ESTIMATE package (35) was employed to evaluate the immunological activity of the tumor. ESTIMATE quantitatively analyzes the immune activity of a tumor sample according to its gene expression profile to obtain an immune score per tumor sample. Herein, the discrepancies in immune infiltration features between the two groups of patients with COAD were compared.

### 2.10 Analysis of copy number variation (CNV)

To compare the copy number differences between the two groups of TCGA-COAD patients, the TCGAbiolinks package (36) of R was used to obtain the Masked Copy Number Segment information. The downloaded CNV fragments were subjected to GISTIC 2.0 analysis by GenePattern (https://cloud.genepattern.org); default parameters were used for the analysis.

### 2.11 Establishment of the prognostic model according to the NPs risk score

The predictive power of the NPs risk score in combination with clinicopathological characteristics on OS based on univariate and multivariate Cox analysis was used to demonstrate that the NPs risk score in combination with clinicopathological features can be used to estimate patient prognosis. The risk scoring model was then combined with clinicopathological features to establish a nomogram, and the accuracy of the model was reflected by the AUC values under the time-ROC curve. The performance of the rosette was assessed using a calibration curve that compared the predicted values of the rosette with the observed actual values. Testing of the model was carried out using the bootstrap method, and internal re-sampling was performed 1000 times.

### 2.12 Establishment of the PPI network and screening of hub-genes

The STRING (37) online tool was applied to establish the PPI network. Genes with scores > 0.7, which indicates high credibility, were selected from the STRING database to construct the network model visualized using Cytoscape (version3.7.2) (38). The Maximal Clique Centrality (MCC) of each node was calculated using the cytoHubba plug-in (39) in Cytoscape software.

### 2.13 Establishment of the ceRNA network according to mRNA-miRNA-lncRNA

Information on the miRNA-mRNA interactions was collected from the miRTarBase database (40). The core mRNAs acquired from the PPI analysis were used to predict the miRNAs that might be regulated. The relevant lncRNAs were further predicted based on evidence from the luciferase reporter gene assay. The results of ceRNA analysis were visualized using Cytoscape software. The P values of all hypothetical tests were two-sided, and a p value of less than 0.05 was considered to indicate statistical significance.

### 2.14 Statistical analysis

Data analysis was performed using R software. version 4.0.2. Independent Student’s t test and Mann–Whitney U test (namely Wilcoxon rank-sum test) were used to estimate the differences between two groups of normally distributed and two groups of non-normally distributed continuous variables, respectively. The χ2 test or Fisher exact test was carried out to determine the difference between the two groups of categorical variables. Survival analysis was carried out using R’s survival package. The Kaplan–Meier survival curve was applied to display survival differences, and log-rank test was performed to compare the differences in survival. Univariate and multivariate Cox analyses were based on the R survival package. LASSO analysis was carried out using the glmnet R package (41).

## 3 Results

### 3.1 Expression and mutation of necroptotic genes in CC patients

**Figure 1:**
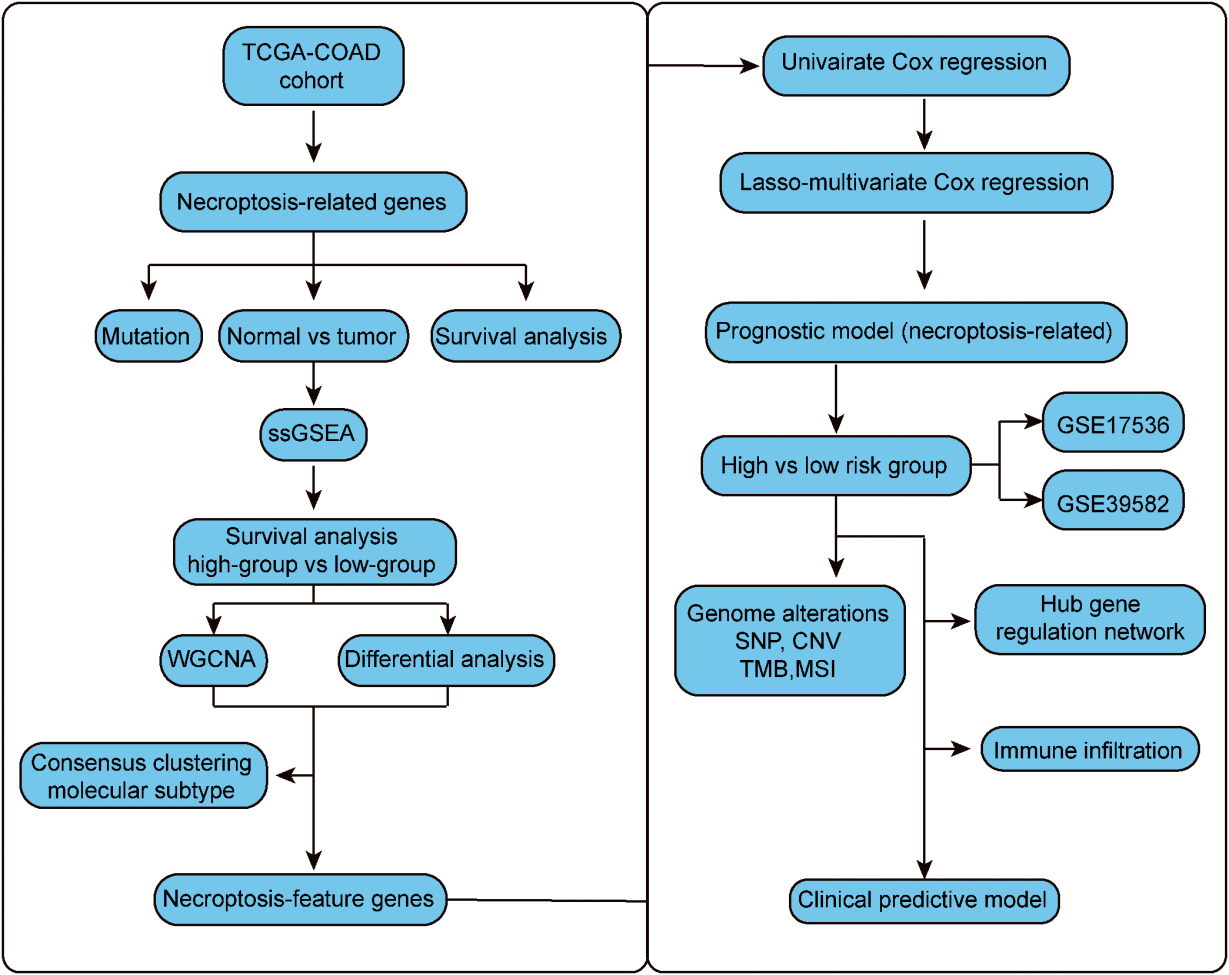
The flow chart.

**Figure 2:**
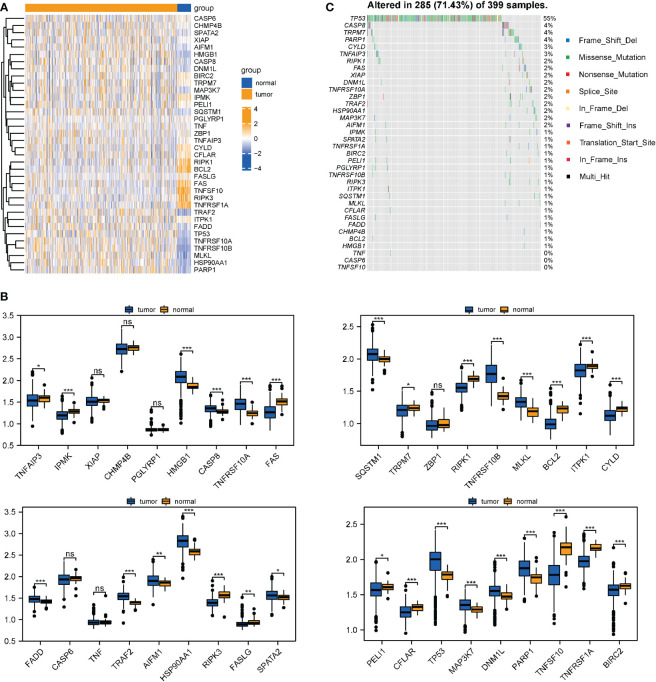
Differential expression and mutation information of necroptotic genes in TCGA-COAD datasets. **(A, B)** Necroptotic genes were compared between the normal and tumor groups in TCGA-COAD dataset using the Wilcoxon test. *P < 0.05, **P < 0.01, ***P < 0.001. **(C)** Mutation information of the necroptotic genes in TCGA-COAD. "ns" represents "no significance".

The whole research design was illustrated in **Figure 1**. First, 36 necroptotic genes were extracted from the RNA-seq data of TCGA-COAD and their expression differences were compared between the normal group and tumor group. A total of 30 necroptotic genes were found to be differentially expressed, and only 6 genes were not differentially expressed. Such results suggest that necroptosis may play crucial role in COAD (**Figures 2A, B**). According to the somatic mutation data of TCGA-COAD samples, mutation information was obtained for the 36 necroptotic genes using the maftools package. The necroptotic genes were not found to mutate significantly in COAD patients, except TP53, in which the mutation frequency of TNF, CASP6, and TNFSF10 was less than 1% (**Figure 2C**). In addition, the prognostic status of the 36 genes was analyzed. Only TRAF2, RIPK3, and IPMK genes were found to have significant prognostic differences, and may thus serve as potential prognostic markers (**Figure S1**).

### 3.2 Calculation of the necroptosis score and screening of characteristic genes

**Figure 3:**
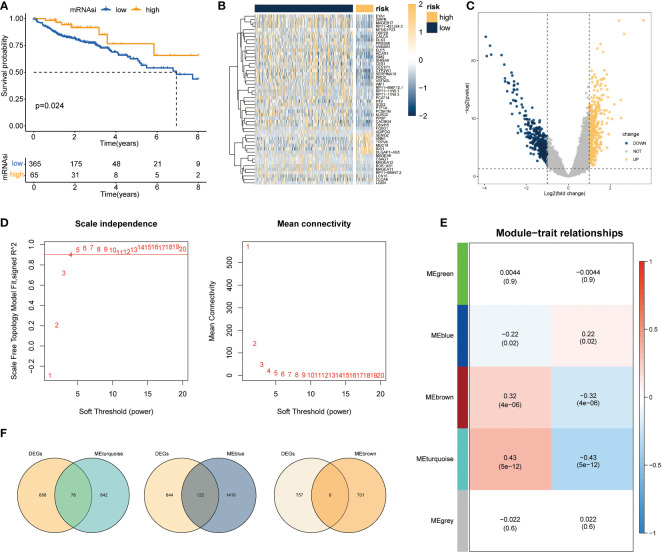
Screening of genes associated with necroptosis. **(A)** Survival analysis results revealed marked difference in survival status between groups with high and low necrotizing apoptosis scores (log-rank *P* =0.024); **(B, C)** Volcano maps and heat maps revealing DEG expression among COAD samples in the groups with high and low necrotizing apoptosis. **(D)** Quality control result selected by WGCNA softpower as 4. **(E)** Set of genes associated with the necroptosis phenotype analyzed and screened using WGCNA. The heat map demonstrated the correlation and significant difference between different gene modules and necroptosis score, where the P values are shown in parentheses. **(F)** Intersection of DEGs and genes in the significant module of WGCNA.

Based on the above differential necroptosis genes, the NPs of each COAD patient was obtained using the ssGSEA algorithm to represent the necroptosis level of the patient. The optimal cut-off value was determined using prognostic analysis based on the necroptosis score. Thereafter, TCGA-COAD samples were divided into groups with high NPs and low NPs. Survival analysis revealed that patients with low necrosis apoptosis scores had worse prognosis than patients with high necroptosis score (log-rank *P* < 0.024; **Figure 3A**). Subsequently, 766 DEGs were acquired through rigorous analysis, including 380 significantly upregulated and 386 downregulated genes, respectively (**Figures 3B, C**). A gene co-expression network was also established to identify biologically significant gene modules through WGCNA and further identify genes closely related to COAD necroptosis. In this study, 4 modules (except grey module) were obtained for subsequent analysis (**Figures 3D, E**). As shown in **Figure 3F**, we integrated the difference analysis results with the MEturquoise, MEblue, and MEbrown modules to obtain a total of 209 necrotizing apoptotic characteristic genes.

### 3.3 Identification of necroptotic subtypes

Based on the above genes with necroptosis characteristics, consistent clustering was employed to cluster LIHC samples. Here, K=2 was selected and two subgroups, subgroup 1 and subgroup 2, were obtained (**Figure S2A**). Dimension reduction analysis was conducted *via* PCA and the PCA results of the two groups were plotted. Based on the results, the degree of differentiation between the two groups was not obvious, which may be due to the insignificant clustering gene characteristics, resulting in insignificant grouping differences (**Figure S2B**). The prognostic characteristics of subgroup 1 and subgroup 2 was subsequently analyzed using the KM curve; however, no distinct difference in prognosis was found between the two groups, suggesting that clustering subtypes based on all necroptotic characteristic genes could not distinguish differences in the prognosis of patients (**Figure S2C**). However, when the expression distributions of characteristic genes of type 1 and type 2 were compared, necroptotic characteristic genes were found to be significantly differentiated in the two subtypes, suggesting that the classification is of guiding significance for assessing the mechanism of necroptosis, but not suitable for identifying clinical prognostic markers (**Figure S2D**).

### 3.4 Construction and evaluation of risk models related to necroptosis

**Figure 4:**
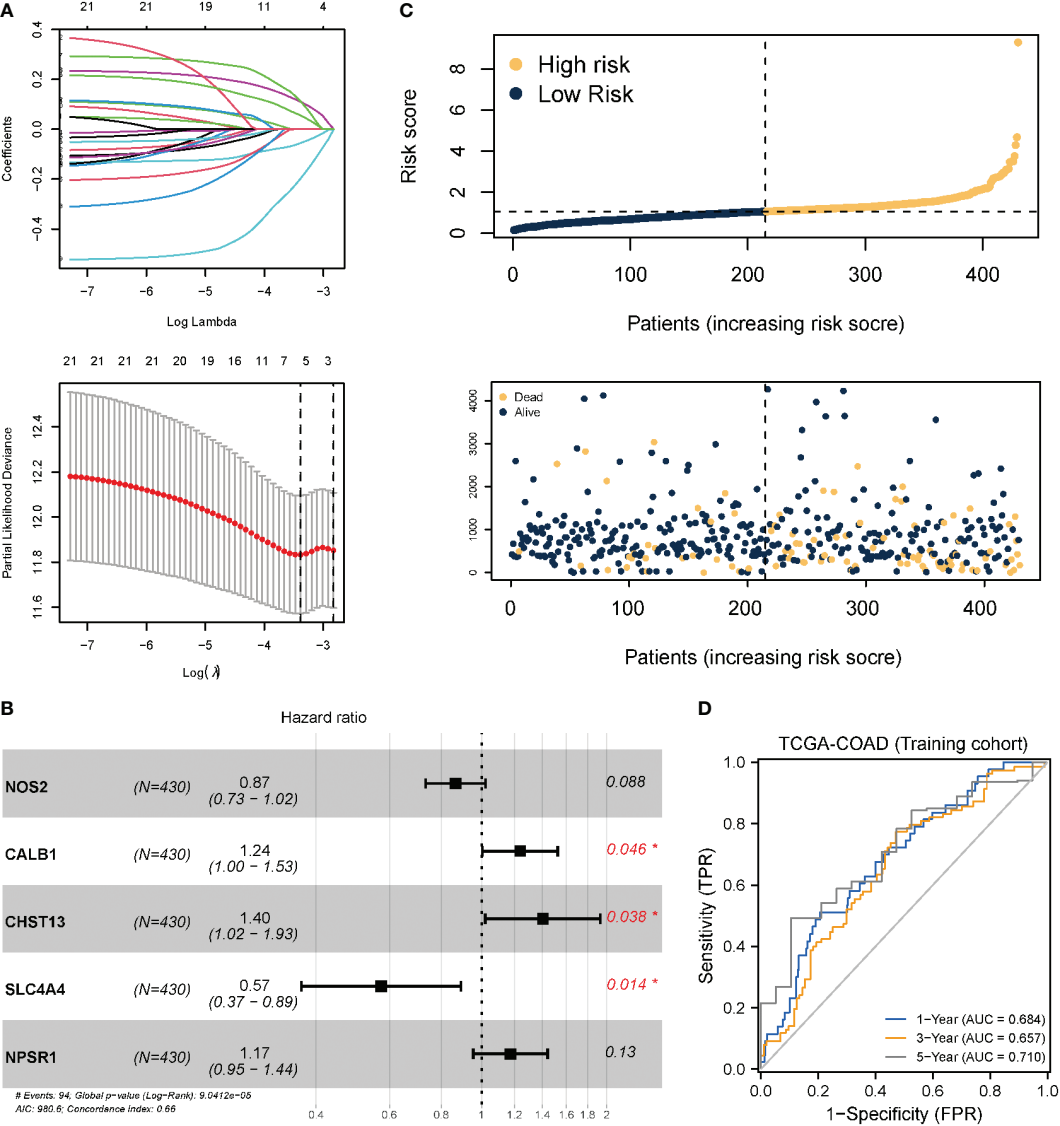
Prognostic model and model test based on necroptotic characteristic genes. **(A)** LASSO regression analysis; the number of variables corresponding to the optimal λ value is 5. **(B)** Three genes identified as independent prognostic factors through multivariate Cox stepwise regression analysis. *P < 0.05. **(C)** Risk score distribution and survival status of COAD patients; **(D)** TimeROC curve of TCGA-COAD (training set). Internal validation was performed using the Bootstrap method, with 1000 iterations.

Based on the necroptosis characteristic genes, a necroptosis-related risk score system was constructed to quantitatively evaluate the prognostic information of each COAD patient by risk score. First, univariate Cox regression analysis revealed that 23 genes met the screening criteria (*P* < 0.05). Dimension reduction was analyzed using LASSO (**Figure 4A**). When 5 variables were present, the most stable model was obtained. Multivariate Cox regression analysis revealed that Calbindin 1 (CALB1), Carbohydrate sulfotransferases (CHST13), and solute carrier family 4 member 4 (SLC4A4) were independent prognostic factors (**Figure 4B**). Multivariate Cox analysis was also carried out to obtain the model coefficients of important characteristic genes. Thereafter, the gene expression was multiplied and summed with its coefficients to construct a risk score. The final risk score (necroptosis risk score related to prognosis) was calculated for each sample. In terms of the risk score and gene expression values of patients, a heat map of the risk factors was plotted to show the distribution of the risk score (**Figure 4C**). The time-dependent ROC curve analysis revealed AUC values of 0.684, 0.657, and 0.710 for the 1-, 3-, and 5-year OS, respectively, which indicated that risk score was an ideal predictor of OS in COAD patients (**Figure 4D**). For the external dataset test, the GSE39582 and GSE17536 datasets were employed. After data normalization, the model was tested. For GSE39582, the ROC curve revealed AUC values of 0.682, 0.623, and 0.708 for the 1-, 3-, and 5-year OS, respectively. For GSE17536, the AUC values were 0.665, 0.712, and 0.758 for the 1-, 3-, and 5-year OS, respectively, indicating that the model had been well tested in external datasets (**Figure S3**).

### 3.5 Analysis of DEGs and functional enrichment in patients with high- and low-risk necroptosis score

**Figure 5:**
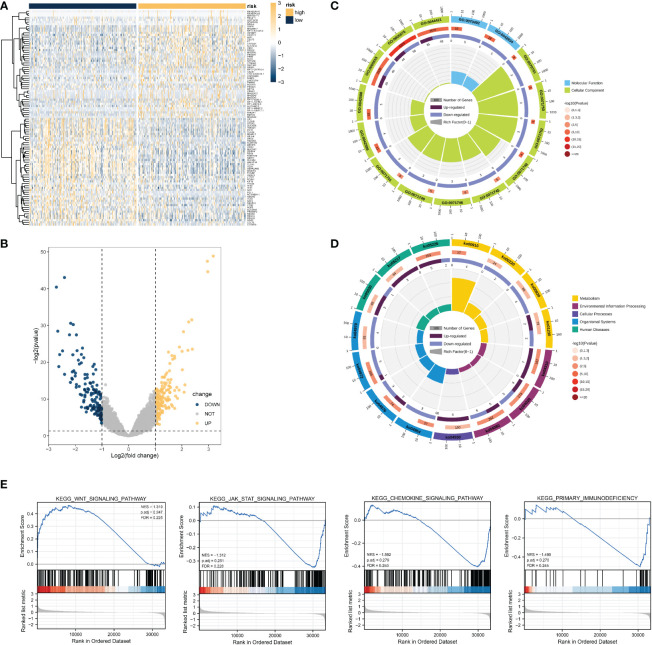
DEG analysis and functional enrichment analysis based on the necroptosis-related risk model. **(A, B)** Volcano map and heat map revealing DEG expression between the high- and low- risk groups in TCGA-COAD dataset. **(C)** GO analysis revealed that the differential genes were correlated with GO:0044421 extracellular region part, GO:0005576 extracellular region, GO:0042588 zymogen granule, GO:0071752 secretory dimeric IgA immunoglobulin complex, and other functions. **(D)** KEGG results revealed that these DEGs participated in nitrogen metabolism, bile secretion, rheumatoid arthritis, and other pathways. **(E)** GSEA results suggested that the KEGG results were similar to those of differential gene enrichment, and the main enrichment pathways were the WNT pathway, JAK-STAT pathway, immune-related pathway, etc.

To determine the role of the necroptosis-related risk model on the evolution of COAD samples, TCGA-COAD patients were divided into high-risk group and low-risk group based on the expression median value of the COAD patient risk model score in TCGA dataset. Subsequently, the DEGs in the two groups of patients were identified. Overall, 317 genes were significantly differentially expressed in COAD patients, among which 185 and 132 genes were significantly upregulated and downregulated, respectively (**Figures 5A, B**).

Functional enrichment analysis was performed using 317 genes identified as significantly DEGs. The GO analysis results revealed that the significant DEGs were related to GO:0044421 extracellular region part, GO:0005576 extracellular region, GO:0042588 zymogen granule, GO:0071752 secretory dimeric IgA immunoglobulin complex, and other functions (**Figure 5C**). KEGG functional analysis suggested that significant DEGs mainly had an impact on nitrogen metabolism, bile secretion, and rheumatoid arthritis, and other pathways (**Figure 4D**). Many pathways were found to be related to immunity, such as the chemokine signaling pathway, WNT signaling pathway, etc. Detailed GO and KEGG results are presented in **Tables S2** and **S3**.

Based on the results of expression analysis, we continued GSEA and summarized the related results of the pathway database based on C2. KEGG pathway results and GSEA results revealed distinct differences in the activity of the JAK-STAT signaling pathway, WNT signaling pathway, fructose and mannose metabolism, primary immunodeficiency, and nitrogen metabolism (**Figure 5**). The detailed results of GSEA and the metabolism-related pathways are provided in **Table S4**. These findings coincide with those obtained from the KEGG database, and suggest the activation and inhibition characteristics of the high- and low-risk groups.

### 3.6 Protein interaction and regulatory network analysis

**Figure 6:**
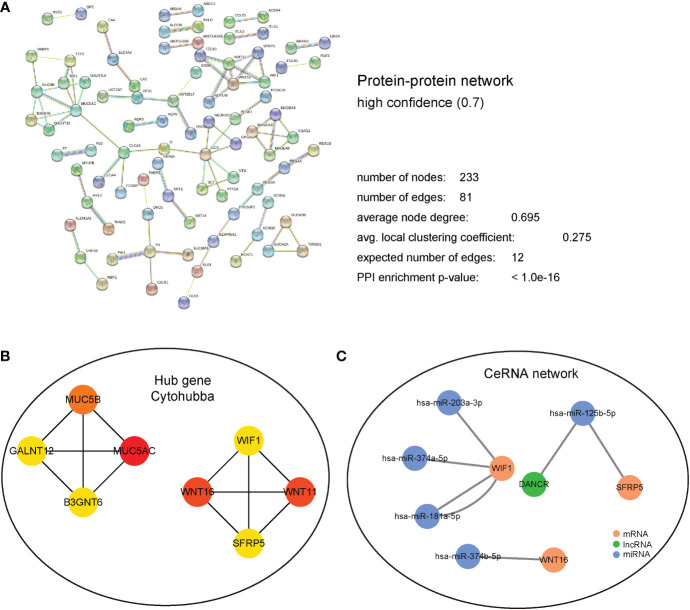
PPI and regulatory network analysis. **(A)** PPI regulation network, detailed display of the network node information, connection line information, and the composition of the different sub-network information. **(B)** Hub gene regulation network based on cytoHubba calculation. **(C)** CeRNA regulatory network predicted using the miRTarBase database. Blue represents miRNA; green represents LncRNA; and Brown represents mRNA.

In terms of the DEGs in the high- and low- risk groups, we aimed to identify the hub gene that played a key role, and its potential molecular interaction mechanism. First, the STRING database was used to analyze the protein interaction mechanism. As shown in **Figure 6A**, after screening with a confidence of 0.700, the number of PPI nodes (protein) was 233. Further, 81 edges were identified, with an average connection degree of 0.695 for each node. The enrichment statistic P value of the whole PPI network was less than 1.0e-16.

The interacting proteins were further identified as hub genes using the cytoHubba plugin in Cytoscape. After the calculations, MUC5AC, MUC5B, WNT16, WIF1, and other interacting proteins that had the top 8 scores were found (**Figure 6B**). Subsequently, miRNA molecules and lncRNAs that potentially regulate these hub genes were analyzed using miRTarBase database. Finally, a ceRNA regulatory network was established using Cytoscape (**Figure 6C**).

### 3.7 Differential expression analysis of immunocyte infiltration

**Figure 7:**
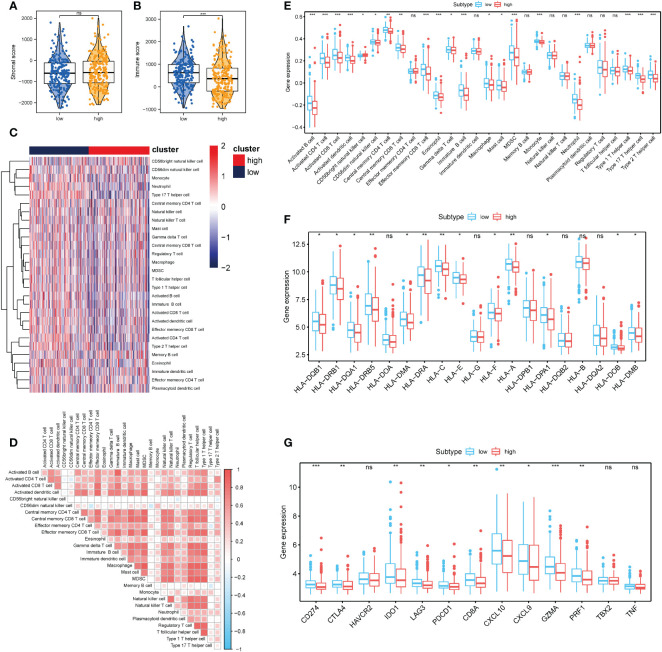
Correlation between necrosis risk scores and the infiltrates of different immunocytes **(A, B)** Immune score and stromal score between the low- and high- risk groups; **(C)** Heat map showing the invasion degrees of 28 different immune cells in TCGA and GEO database; **(D)** Association heat map showing the association between various levels of immunocyte infiltration. **(E)** Differential analysis of 28 different immunocyte infiltration levels between the two groups; **(F)** Analysis of differences in the expression of multiple members of the HLA family between the high- and low-risk related subgroups; **(G)** Differential expression analysis of multiple immunotherapy-related targets between the high- and low-risk related groups. *P < 0.05, **P < 0.01, ***P < 0.001. "ns" represents "no significance".

The influence of necroptosis-associated risk scores in patients with TCGA-COAD on their holistic immune characteristics and varying degrees of immunocyte infiltration was analyzed. Patients in the high-risk group were found to have significantly lower immune-related scores (*P* < 0.001); however, no distinct difference was found in the matrix score (**Figures 7A, B**). ssGSEA was used to appraise the changes and effects of immunological characteristics of COAD tissues during pathogenesis. Through ssGSEA, the relative enrichment scores of 28 different subtypes of immunocytes in the high- and low-risk groups of COAD patients was obtained. Heat maps were generated to illustrate their expression in different patients (**Figure 7C**). Based on the results, the expression abundance of immunocytes in the low-risk group was lower than that in the high-risk group. The correlation analysis results revealed that most immune cell infiltration levels were positively correlated (**Figure 7D**). Differential analysis also revealed distinctions in the infiltration levels of various immune cells between COAD samples in the high and low necrotizing apoptosis-related risk groups. Only effector memory CD8+ T cells, immature dendritic (iDC) cells, and other cells were not found to significantly differ between the groups (**Figure 7E**). A distinct difference was found between the HLA family expression levels and the various immunological targets of the high- and low-risk groups. Further, the immunoactive genes were almost all elevated in the low-risk group (**Figures 7F, G**).

### 3.8 Effects of the necroptosis-related risk score on genomic changes in COAD samples

**Figure 8:**
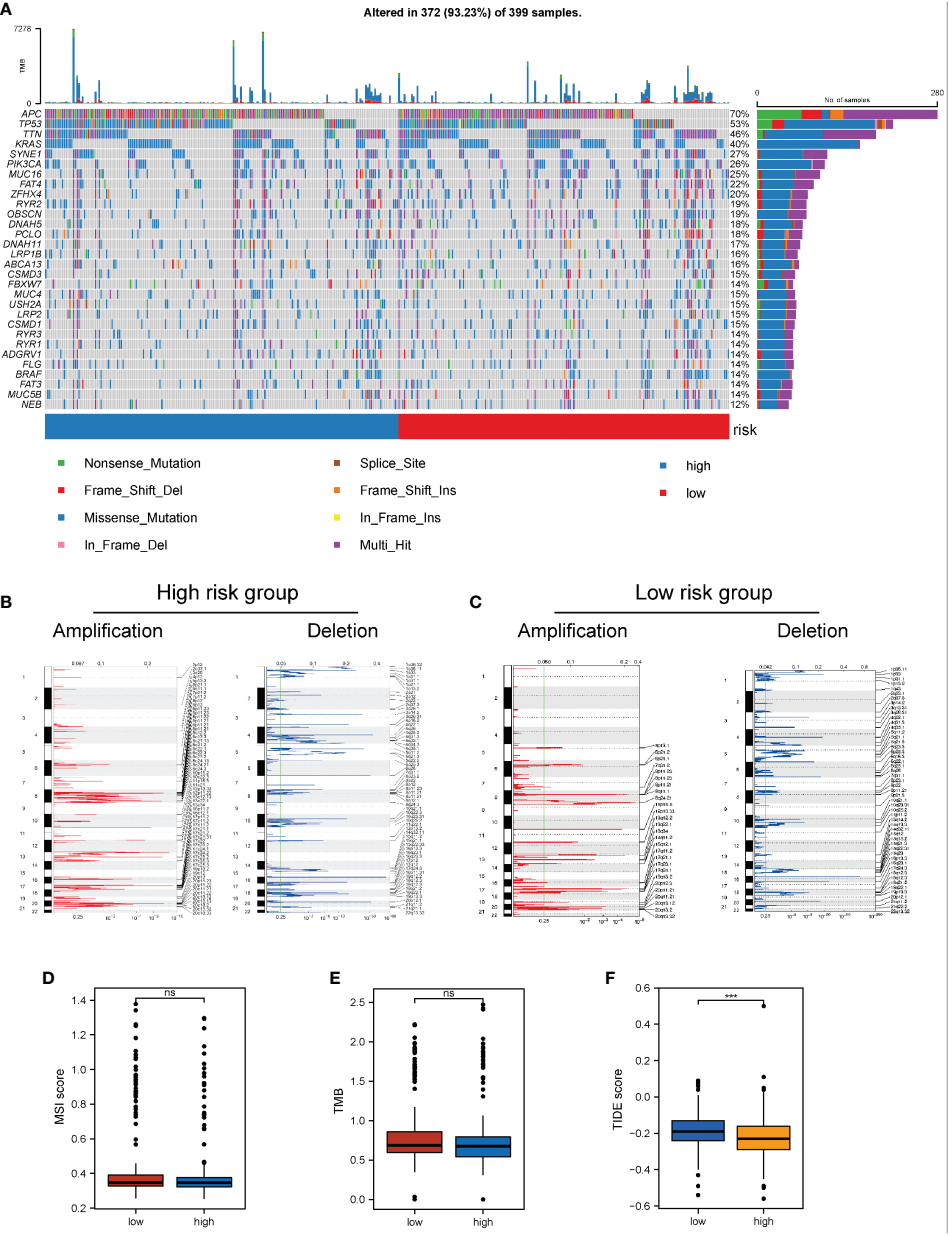
Impact of necroptosis-related risk grouping on genetic variation and immunotherapy in COAD samples. **(A)** Mutation map of common tumorigenic driver genes in patients in the high- and low-risk groups. Mutation information per gene per sample is presented as a waterfall plot, and different colors represent different types of mutation. The subsection above the legend shows the sudden change load; **(B, C)** Changes in the copy number levels of different genes in the high-risk and low-risk groups, where genes with significant copy number increase in red and genes with significant copy number deletions in blue; **(D, E)** Comparison of the difference in MSI level and TMB level between patients in the high- and low-risk groups, respectively; **(F)** Discrepancy between the high-risk and low-risk groups based on the tide score calculated from the tide database. ***P < 0.001. "ns" represents "no significance".

The influence of the necroptosis-related risk score on changes in genetic variation levels, including single nucleotide polymorphism and CNV, in COAD patients was evaluated. The analysis of single-nucleotide mutations in common tumor-driven genes revealed that the high mutation levels were similar or close between patients with high and low scores in the necroptosis-related model (**Figure 8A**). Based on assessments of the frequency of CNV changes, CNV was found to be widely present in high- and low-risk samples. However, no distinct discrepancy in CNV was found between the two groups (**Figures 8B, C**). When TMB and MSI were compared between the two patient groups, no distinct discrepancy in TMB and MSI was found between the high and low risk groups. This result suggests that changes at the genomic level were not significant in the two groups (**Figures 8D, E**).

Based on the significant role of immunotherapy in tumors, the TIDE algorithm was employed to calculate the sensitivity of patients in the high- and low-risk groups to immunotherapy. The TIDE score in the low-risk group was higher than that in the high-risk group, suggesting that the immunotherapy response of the high-risk group might be better than that of the low-risk group (**Figure 8F**).

### 3.9 Establishment of a prognostic model according to the necroptosis-related risk score

**Figure 9:**
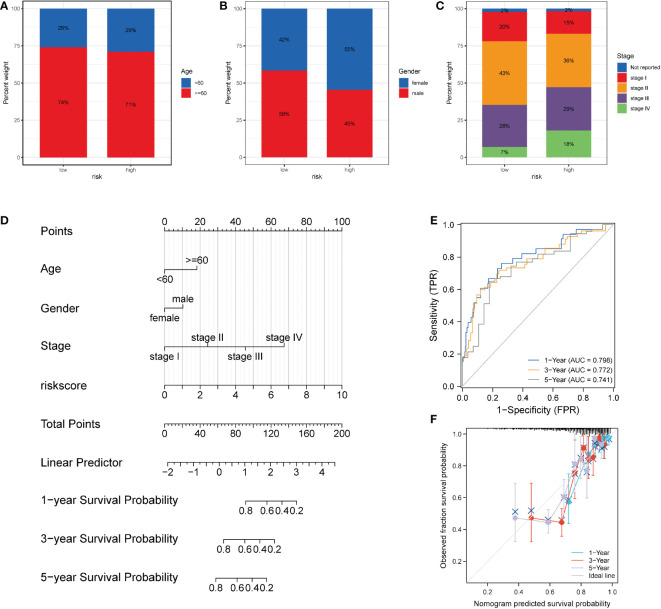
Performance of the necroptosis risk scores in the prediction of prognosis for patients with COAD. **(A-C)** Superimposed histogram showing the proportion of age, sex, and stage in patients in the high- and low-risk groups. The effect of age was similar in both groups, with an increased proportion of women in the high-risk group and significantly more advanced patients in the high-risk group. **(D)** Nomogram of the model. **(E)** Time-dependent ROC curve of the clinical prediction model based on risk score. **(F)** For the calibration curve of the nomogram, the bootstrap method was adopted, and resampling was performed 1000 times. The abscissa is the survival predicted by the nomogram, and the ordinate is the actual observed survival. The calibration plot revealed that the bias-corrected line for 1-, 3-, and 5- years OS was close to the ideal line, indicating good consistency between the predicted value and the actual value.

To further probe the clinical value of necroptosis-related risk score, the clinical characteristics related to the high-risk and low-risk groups, such as the discrepancy in age and TNM stage, were analyzed. Notably, no distinct discrepancy was found in the age of patients in the high-risk group (**Figure 9A**). For gender, the proportion of women in the high-risk group increased (**Figure 9B**). In terms of stage, a significantly higher proportion of advanced patients was identified in the high-risk group (**Figure 9C**). Subsequently, based on the risk scores associated with necroptosis and clinicopathologic features (age and TNM stage), we established a prognostic model for COAD patients (**Figure 9D**) and analyzed the model *via* 1000 resampling using the bootstrap method. Based on timeROC, the AUC values were 0.798, 0.772, and 0.741 for 1-, 3-, and 5- years, respectively (**Figure 9E**). Calibration curves were generated to present the consistency of the model. A good consistency was found between the model’s estimated 1-, 3-, and 5-year OS and the actual observed OS of patients (**Figure 9F**).

## 4 Discussion

In recent years, the incidence of CC among young people has gradually increased (42). Necroptosis has different functions in diverse tumors, including promoting tumor progression in lung cancer, pancreatic cancer, and glioblastoma (43–45), or inhibiting tumor growth in gastric cancer (GC), head and neck squamous cell carcinoma, melanoma and CRC (46–49). Necroptosis also has a two-way effect of promoting cancer and suppressing cancer in breast cancer (50, 51). As a result, we cannot appraise the prognosis of CC according to the expression of individual necrosis regulators alone. Targeting NRGs is regarded as one of the effective methods for reducing tumor chemotherapy resistance, opening up a new approach for cancer treatment (52). A prior study revealed the construction of a prognostic model of lncRNA associated with GC necroptosis to differentiate hot and cold tumors of gastric carcinoma, to ultimately predict prognosis and the effectiveness of immunotherapy (53). Nevertheless, the theory of necroptosis in CC remains indistinct. In this study, prognostic risk models based on NPs characteristic genes was constructed to predict prognosis and immunotherapy, and systematically analyze the correlation between immune cell infiltration, immune checkpoints, and CC.

A total of 30 necroptotic genes were found to be differentially expressed. Thereafter, an analysis of DEGs revealed 766 DEGs in the high NPs group and low NPs group. A gene co-expression network was also established to identify biologically significant gene modules through WGCNA. Finally, a total of 209 necrotizing apoptotic characteristic genes were identified. The results of univariate Cox regression analysis, LASSO, and multivariate Cox regression analysis revealed that CALB1, CHST13, and SLC4A4 are independent prognostic marks. The final risk score was then calculated for each sample.

CALB1 is a vitamin D-dependent calcium-binding protein with six EF hands on the long arm of chromosome 8 at position 21.3 (54). CALB1, a component of Calbindin, has been confirmed to restrain tumor cell apoptosis. A prior study suggested that CALB1 may exert carcinogenic effects in ovarian cancer by inhibiting the p53 pathway (55). CALB1 is overexpressed in nonsmall cell lung cancer (NSCLC) tissues, and has a significant connection with lymph node metastasis and prediction of worse survival (56). In osteosarcoma, the downregulation of CALB1 gene expression resulted in reduced cell proliferation and cell clonal formation (57). In this study, CALB1 was verified to be an independent risk factor for prognosis. Further, its expression was found to increase, indicating poor prognosis of patients. Previous studies did not directly explain the relationship between CALB1 and CC. However, this study provides ideas for future diagnosis and treatment using *CALB1* as an oncogene.

Chondroitin sulfate (CS) is a glycosaminoglycans (GAGs) that participates in multiple biological processes and exerts crucial function in the interaction among stromal tumor cells (58). CHST13 gene is located on chromosome 3q21.3. A prior study suggested that CHST13 may serve as a negative regulator of HCC cell invasion and chemotherapy sensitivity by modulating Mitogen-Activated Protein Kinase (MAPK) activity (59). The mRNA expression of CHST13 was found to be significantly higher in in ovarian cancer specimens than in non-malignant tumor specimens (60). The results of this study indicate that CALB1 is an independent prognostic marker that plays the role of an oncogenic gene in the occurrence and development of CC. Thus, CALB1 could serve as an original biomarker for the diagnosis and prognosis evaluation of CC. However, no prior study has revealed that CHST13 can serve as a high-risk independent prognostic factor for OS. Accordingly, the present study is an important supplement to this field.

*Homo sapiens* solute vector family Member 4 (SLC4A4) is a member of the solute vector family which encodes an electrogenic Na+/HCO3− cotransporter (61). A previous study showed that SLC4A4 is increasingly expressed in prostate cancer tissues and cell lines. Further, the SLC4A4 expression level in cancer tissues was significantly associated with the degree of disease progression. SLC4A4 promotes prostate cancer progression through the Akt-mediated signaling pathway (62). Mir-222-3p expression was increased in PTC, while that of SLC4A4 was low. SLC4A4 could reverse the promoting function of Mir-222-3p on the proliferation, invasion, and migration of PTC cells (63). SLC4A4 had a lower expression in CRC than normal tissue, indicating that SLC4A4 was associated with poor prognosis (64). This study revealed that SLC4A4 may be an individual prognostic factor for CC patients and may exert a protective function in the tumorigenesis and progression of CC, which aligned well with the proposals from existing studies.

TCGA-COAD samples were divided into high- and low-risk groups based on the median expression value of the COAD patient risk model score in TCGA dataset. DEG analysis and functional enrichment analysis were then performed. The main enrichment pathways included the WNT pathway, JAK-STAT pathway, primary immunodeficiency pathway, chemokine pathway, fructose and mannose metabolism, nitrogen metabolism, etc.

The abnormal WNT signaling pathway is highly relevant to tumorigenesis and progression of multiple tumors, including CC (65–67). A previous study confirmed that activation of Wnt/β-catenin signaling contributes to the aberrant expression of several oncogenes that regulate the dedifferentiation phenotype and EMT in CC cells (68). Another study demonstrated that RBBP4 activates the Wnt/β-catenin pathway to accelerate the progression of CC (69). Based on this analysis, the WNT signaling pathway was identified to be significantly differentially enriched in the high-risk group phenotype.

The JAK/STAT pathway plays an increasingly vital role in regulating immune function, cell proliferation, differentiation, and death (70–72). Fibroblast growth factor receptor was reported to mediate PD-L1 expression in CC by activating the JAK2/STAT3 signaling pathway (73). Notably, activation of the JAKs structure promotes phosphorylation of the STAT family (74). The STAT3 signaling pathway is in close contact with the construction of a tumorigenic inflammatory microenvironment (75). The proliferation and viability of macrophages were reported to be enhanced by STAT3 activation, the immune tolerance of CC cells, and inhibition of extracellular matrix remodeling, thereby playing tumor-promoting roles (76). Based on this analysis, the JAK/STAT signaling pathway was identified to be significantly differentially enriched in the low-risk group phenotype. Consistent with the results of this study, necroptosis may promote tumor progression by inhibiting the JAK/STAT pathway.

A systematic review of all cases of clinically diagnosed primary immunodeficiency and early-onset gastrointestinal (GI) cancer in three publicly available databases (MEDLINE, SCOPUS, and EMBASE) was previously conducted. Based on the results, primary immunodeficiency may be linked with potential risk factors for GI tumor. Adenocarcinomas of the stomach and colon were identified as the most common GI tumor (77). A previous literature revealed the involvement of chemokine (CC theme) ligand 7 (CCL7) in the progression of CRC (78). Another literature revealed the ectopic expression of the novel chemokine, CXCL17, in primary CC. The expression of CXCL17 might inform the prognosis of CC patients as CXCL17 enhances angiogenesis and attracts immune cells (79).

Based on the differential genes in the high and low risk groups, we opted to identify the key hub genes and their underlying molecular interaction mechanisms. These hub genes were *MUC5AC*, *MUC5B*, *WNT16*, *WNT11*, *WIF1*, *SFRP5*, *B3GNT6*, and *GALNT12*.

Mucin is a type of high molecular weight glycoprotein that is mainly involved in protecting epithelial cells of different organs from physical, chemical, and pathogenic damage (80). Mucin has abnormal expression in many malignant tumors, which is correlated with the proliferation, migration, invasion, adhesion and metastasis of tumor cells (81, 82). Changes in mucin expression have been reported to have a high correlation with the occurrence of CRC (81). Normally, expression of the secreted mucin, MUC5AC, is restricted to the stomach, lung, ear, conjunctiva, nasopharynx, and gallbladder. Several studies revealed that secreted MUC5AC is overexpressed in pancreatic cancer, lung cancer, and breast cancer (83–85). In fact, the secreted mucin, MUC5AC, was not identified in normal colonic mucosa, but was present in benign and malignant colon (80, 86). Prior literature confirmed that MUC5AC across the membrane protein, CD44, mediated the initiation and progression of CC, and provided resistance to chemotherapy in CRC through the β-catenin/p53/p21 signaling pathways (87). Secreted MUC5B mucin is generally not expressed in normal adult gastrointestinal mucosa, but has been proven to be differentially overexpressed in some subtypes of GC and CRC (88–90).

Numerous studies proved that over-activation of the Wnt signaling pathway is the main culprit in the onset of most human malignant tumors (91, 92). The Wnt signaling pathway plays a crucial role in multiple biological processes, such as embryogenesis and tissue homeostasis, exerting significant functions in the tumorigenesis and progression of CRC (93). A previous study found one or more mutations downstream in the Wnt signaling pathway, especially adenomatous polyposis coli (APC), in more than 90% of patients with CRC (94). WNT16 is one of the most impressive members of the WNT pathway (95, 96). The Wnt signaling pathway consists of canonical signals and noncanonical signals. Transmembrane proteins and their receptors mediate canonical Wnt signaling. Atypical Wnt signaling involves two pathways: the Wnt/Ca2+ pathway and the Wnt/c-jun N-terminal kinase (JNK)(planar cell polarity) pathway (97, 98). Wnt11 exerts its role *via* the noncanonical WNT pathway (97). Studies have confirmed that Wnt11 has a vital effect in the regulation of CRC cell proliferation, migration, and invasion (99, 100).

Wnt inhibitory factor 1 (WIF1) can interact with the Wnt protein to inhibit the canonical and non-canonical Wnt pathways to exert tumor inhibitory effect. WIF1 silenced by methylation has been found to participate in CC progression (101).

Secreted frizzled-related protein 5 (SFRP5) is a new type of adipocytokine, belonging to the SFRP family. Plasma SFRP5 levels were found to be distinctly decreased in obese patients and patients with diabetes, coronary artery disease, and other related diseases (102, 103). SFRP5 is underexpressed in moderate tumor tissues including lung cancer, ovarian cancer, GC, and breast cancer tissues, and is associated with poor prognosis (104–107). GALNT12 has been revealed to be a strong candidate for CRC susceptibility (108).

The B3GNT protein family is differentially expressed in multiple cancers, such as GI cancer, pancreatic carcinoma, and prostate cancer (109–111). The expression of B3GNT was found to be significantly decreased in GC and CRC (112). Although the 8 hub genes are relevant to tumorigenesis and progression, relevant studies on CRC are insufficient.

Based on increasing evidence, the ceRNA regulatory network plays a key role in the progression of various common cancers (113, 114). Shang et al. (115) found that the tumor-derived exosome, circPACRGL, acts as a sponge molecule of miR-142-3p/miR-506-3p, promoting the propagation, diversion, invasion, and adhesion of CC cells and N1 to N2 neutrophil differentiation. Wu et al. (116) showed that the LNC473-MIR574/miR15B-APAF1 IRES signaling axis could manipulate the propagation and apoptosis of CRC cells to influence the initiation and progression of CRC. In this study, a ceRNA regulatory network was constructed with 1 lncRNA, 5 miRNAs, and 3 mRNAs, revealing the potential regulatory mechanism of lncRNA-miRNA-mRNA in CC, and indicating the direction for further exploration of the pathogenesis of CC.

The immune microenvironment of CC and immunotherapy for CC patients should be explored. Immune cells in the TME perform vital functions in tumor progression (117). Based on prior studies, immune checkpoint inhibitors (ICIs) have great potential in immunotherapy of CC (118).

Immunocheckpoint inhibitor therapy of CC is in the “MSI era” because microsatellite instability (MSI) or mismatch repair gene status (MMR) is the best predictor of efficacy. Based on MSI status, CC patients can be divided into two groups according to the efficacy of immunotherapy: “advantaged population” – MSI-H/dMMR type (MSI-H type for short); “Invalid population” – MSS/pMMR type cancer (MSS type cancer for short) (119). However, only about 5% of metastatic colorectal cancer (mCRC) is MSI-H, and about 95% is MSS type (120). How to turn “cold tumor” into “hot tumor” effective for immunotherapy has been a hot research direction. EYNOTE 016 Phase II clinical trial results showed that the objective response rate (ORR) of MSI-H mCRC patients was 40%, while the ORR of MSS mCRC patients was 0 (121). Immunotherapy has enriched the treatment modalities of multiple malignancies, including cytotoxic T lymphocyte-associated protein 4 (CTLA-4) and inhibitor of programmed death-1 (P D-1)/programmed cell death ligand 1(PD-L1). In patients with MSS mCRC, single-agent immunotherapy has failed, and multiple clinical trials of immunotherapy in combination with other therapies are being actively explored, including combination immunotherapy and immunotherapy combined with targeted therapy, radiotherapy, oncolytic virus, bisspecific antibodies, etc. Pd-1/PD-L1 inhibitors in combination with other immunotherapies may play a synergistic role in enhancing the antitumor effect. In a Phase II trial, durvalumab, a PD-L 1 inhibitor, combined with CTLA-4 inhibitor tremelimumab in refractory mCRC patients (92% pMMR/MSS) showed significant benefits in overall survival (OS) (122). In addition, in 2019, 24 patients with pMMR/MSS colon cancer who had failed standard treatment were included in the REGONIVO study. The ORR reached 33.3% after treatment with regorafenib combined with navulizumab, which significantly improved progression free survival (PFS) and OS (123).

In this study, the low-risk group was found to have higher levels of infiltration of multiple immunocytes, several HLA family members, and multiple immunotherapy targets. In addition, based on the significant role of immunotherapy in tumors, the TIDE algorithm was used to assess the sensitivity of both groups to immunotherapy. The TIDE score was lower in the high-risk group than the low-risk group, suggesting that the immunotherapy response of the high-risk group might be better than that of the low-risk group.

To further enhance the prediction accuracy of the model, a nomogram model based on the risk score prognostic model and clinical indicators (including age and pathological stage), which markedly improved the precision of the model, was established. The time-dependent ROC curve suggested that the risk score had favorable predictive performance for the OS of COAD patients. Calibration curves revealed a good consistency between the model’s estimated 1-, 3-, and 5-year OS and the actual observed OS of patients.

This study had some limitations. First, clinical information and basic experimental verification are lacking. Further, the reliability of the results is dependent on the accuracy of TCGA dataset. In the future, the results of this study should first be verified through clinical trials and basic experiments. Prospective studies are also needed as retrospective studies may be subject to bias. Finally, clinical follow-up data are lacking to prove the accuracy of our prognostic model.

In this study, a prognostic risk model based on NRGs was established, and the diagnostic and predictive significance of the risk model was evaluated. The results of this study will help to reveal the pathogenesis of CRC, enabling the development of new diagnostic ideas, and facilitate the search for new therapeutic targets and prognostic molecular markers.

## Data availability statement

The datasets for this study can be found in the Cancer Genome Atlas (TCGA) Genomic Data Commons (GDC) website (https://portal.gdc.cancer.gov/). The original contributions presented in the study are included in the article/**Supplementary Material**, further inquiries can be directed to the corresponding author.

## Author contributions

WY designed the ideas for this paper. WY, DG, and FM contributed to the writing of the manuscript. SL, LP, ZZ, and YZ contributed to data collation and data analysis. WY, SL, YH, and XC analyzed and interpreted the data. All authors contributed to the article and approved the submitted version.
