# Supplementary material for: Integrated analysis of necroptosis-related genes for evaluating immune infiltration and colon cancer prognosis
Source: Front Immunol. 2022 Dec 22;13:1085038. doi: 10.3389/fimmu.2022.1085038 (PMC9814966; doi:10.3389/fimmu.2022.1085038)
Supplement: Supplementary file 1 [file DataSheet_1.docx]

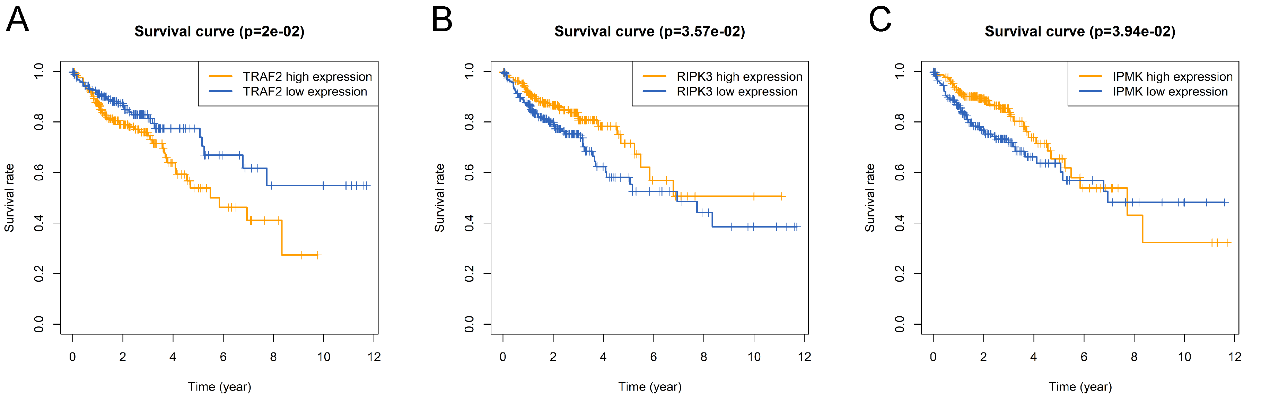


Figure S1. Necroptotic genes with prognostic differences in TCGA-COAD dataset. KM curve revealed that *TRAF2* (A), *RIPK3* (B), and *IPMK* (C) genes had significant survival differences.


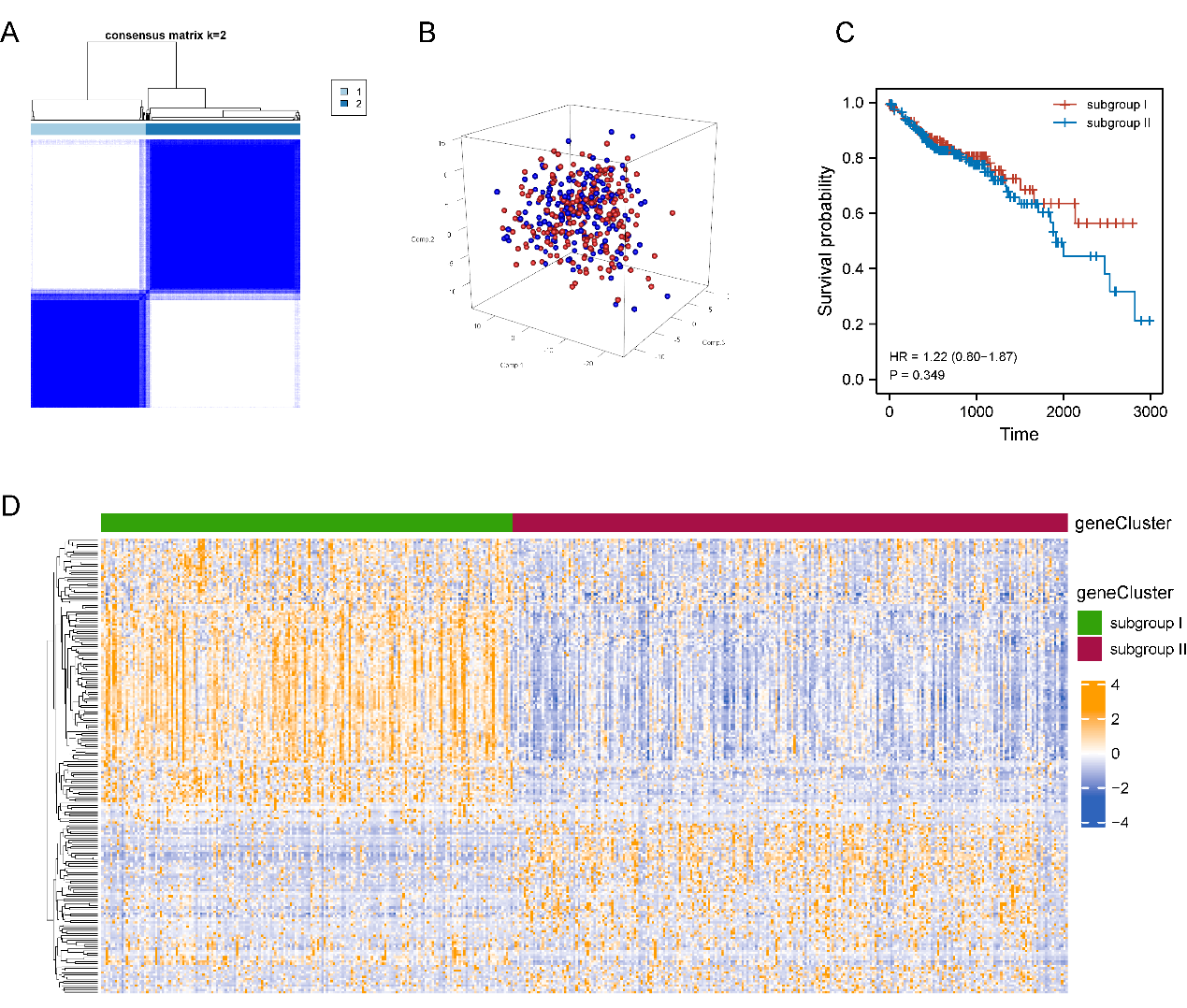


Figure S2. COAD typing based on necroptotic characteristic genes. A: Heat map of the clustered samples at K=2 based on necroptotic characteristic genes using the consistent clustering method. B: 3DPCA diagram of subgroup 1 and subgroup 2; C: KM method for the comparison of the survival difference between subgroup 1 and subgroup 2. D: Heatmap showing necroptosis and expression abundance as the cause in the subgroups.


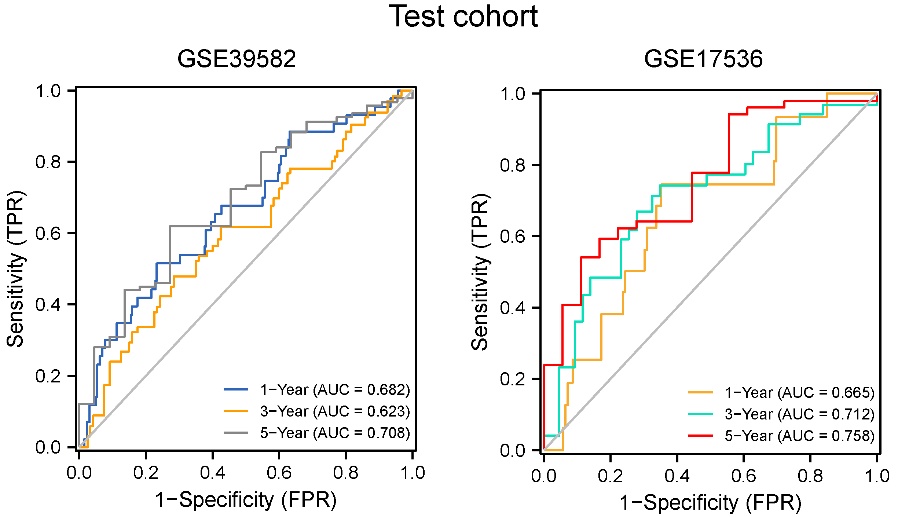


Figure S3. External dataset test of a necroptosis prognostic model. GSE39582 and GSE17536 were the external test sets for the prognostic models.
