# Supplementary material for: Integrated analysis of necroptosis-related genes for evaluating immune infiltration and colon cancer prognosis
Source: Front Immunol. 2022 Dec 22;13:1085038. doi: 10.3389/fimmu.2022.1085038 (PMC9814966; doi:10.3389/fimmu.2022.1085038)
Supplement: Supplementary file 2 [file Table_1.docx]

Table S1. Basic information table of GEO datasets.

| **Dataset** | **Platform** | **Samples** | **Type** | **Reference** |
| --- | --- | --- | --- | --- |
| GSE17536 | GPL570 | 177 | tissue | [18] |
| GSE39582 | GPL570 | 580 | tissue | [19] |

Table S2. Results of GO enrichment analysis.

| **class** | **ID** | **Descrption** | **Pvalue** | **Qvalue** |
| --- | --- | --- | --- | --- |
| Cellular Component | GO:0005576 | extracellular region | 2.20E-12 | 9.31E-10 |
| Cellular Component | GO:0005615 | extracellular space | 4.44E-10 | 9.39E-08 |
| Cellular Component | GO:0044421 | extracellular region part | 1.99E-09 | 2.80E-07 |
| Cellular Component | GO:0042588 | zymogen granule | 7.89E-07 | 8.34E-05 |
| Cellular Component | GO:0070701 | mucus layer | 1.92E-06 | 0.000116 |
| Cellular Component | GO:0071750 | dimeric IgA immunoglobulin complex | 1.92E-06 | 0.000116 |
| Cellular Component | GO:0071752 | secretory dimeric IgA immunoglobulin complex | 1.92E-06 | 0.000116 |
| Cellular Component | GO:0042589 | zymogen granule membrane | 7.30E-06 | 0.000358 |
| Cellular Component | GO:0071748 | monomeric IgA immunoglobulin complex | 7.62E-06 | 0.000358 |
| Molecular Function | GO:0070492 | oligosaccharide binding | 9.56E-07 | 0.000564 |
| Molecular Function | GO:0042834 | peptidoglycan binding | 1.84E-06 | 0.000564 |
| Cellular Component | GO:0071745 | IgA immunoglobulin complex | 1.89E-05 | 0.000614 |
| Cellular Component | GO:0071746 | IgA immunoglobulin complex, circulating | 1.89E-05 | 0.000614 |
| Cellular Component | GO:0071749 | polymeric IgA immunoglobulin complex | 1.89E-05 | 0.000614 |
| Cellular Component | GO:0071751 | secretory IgA immunoglobulin complex | 1.89E-05 | 0.000614 |
| Biological Process | GO:0019730 | antimicrobial humoral response | 1.79E-07 | 0.00062 |
| Molecular Function | GO:0004252 | serine-type endopeptidase activity | 5.96E-06 | 0.001219 |
| Molecular Function | GO:0008236 | serine-type peptidase activity | 1.57E-05 | 0.001964 |
| Molecular Function | GO:0022838 | substrate-specific channel activity | 1.62E-05 | 0.001964 |
| Molecular Function | GO:0017171 | serine hydrolase activity | 1.92E-05 | 0.001964 |
| Molecular Function | GO:0005229 | intracellular calcium activated chloride channel activity | 3.34E-05 | 0.002567 |
| Molecular Function | GO:0061778 | intracellular chloride channel activity | 3.34E-05 | 0.002567 |
| Molecular Function | GO:0015267 | channel activity | 5.34E-05 | 0.00299 |
| Molecular Function | GO:0022803 | passive transmembrane transporter activity | 5.34E-05 | 0.00299 |
| Molecular Function | GO:0015318 | inorganic molecular entity transmembrane transporter activity | 5.45E-05 | 0.00299 |
| Molecular Function | GO:0035240 | dopamine binding | 5.84E-05 | 0.00299 |
| Biological Process | GO:0010817 | regulation of hormone levels | 4.83E-06 | 0.005954 |
| Biological Process | GO:0001101 | response to acid chemical | 6.77E-06 | 0.005954 |
| Biological Process | GO:0044278 | cell wall disruption in other organism | 6.86E-06 | 0.005954 |
| Cellular Component | GO:0031233 | intrinsic component of external side of plasma membrane | 0.000207 | 0.00625 |
| Molecular Function | GO:0030246 | carbohydrate binding | 0.000143 | 0.006442 |
| Molecular Function | GO:0005216 | ion channel activity | 0.000147 | 0.006442 |
| Biological Process | GO:0007586 | digestion | 9.34E-06 | 0.006489 |
| Molecular Function | GO:0070405 | ammonium ion binding | 0.000183 | 0.007073 |
| Molecular Function | GO:0015108 | chloride transmembrane transporter activity | 0.000184 | 0.007073 |
| Molecular Function | GO:0008509 | anion transmembrane transporter activity | 0.000239 | 0.008516 |
| Molecular Function | GO:0005254 | chloride channel activity | 0.00025 | 0.008516 |
| Cellular Component | GO:0016323 | basolateral plasma membrane | 0.000319 | 0.008995 |
| Molecular Function | GO:0015103 | inorganic anion transmembrane transporter activity | 0.000316 | 0.009846 |
| Molecular Function | GO:0004175 | endopeptidase activity | 0.000321 | 0.009846 |
| Biological Process | GO:0006811 | ion transport | 2.25E-05 | 0.011192 |
| Biological Process | GO:0009605 | response to external stimulus | 2.26E-05 | 0.011192 |
| Molecular Function | GO:0015075 | ion transmembrane transporter activity | 0.000392 | 0.011475 |
| Molecular Function | GO:1904768 | all-trans-retinol binding | 0.000431 | 0.012027 |
| Molecular Function | GO:0005215 | transporter activity | 0.000495 | 0.013207 |
| Cellular Component | GO:0098590 | plasma membrane region | 0.000548 | 0.014483 |
| Molecular Function | GO:1901338 | catecholamine binding | 0.000571 | 0.014607 |
| Molecular Function | GO:0005253 | anion channel activity | 0.000606 | 0.014816 |
| Molecular Function | GO:0022857 | transmembrane transporter activity | 0.000627 | 0.014816 |
| Molecular Function | GO:0004089 | carbonate dehydratase activity | 0.000707 | 0.016087 |
| Cellular Component | GO:0044459 | plasma membrane part | 0.000757 | 0.018177 |
| Cellular Component | GO:0031226 | intrinsic component of plasma membrane | 0.000773 | 0.018177 |
| Biological Process | GO:0006820 | anion transport | 4.22E-05 | 0.018329 |
| Cellular Component | GO:0031526 | brush border membrane | 0.000841 | 0.018725 |
| Molecular Function | GO:0034617 | tetrahydrobiopterin binding | 0.000855 | 0.018749 |
| Molecular Function | GO:0005179 | hormone activity | 0.000887 | 0.01879 |
| Biological Process | GO:0002138 | retinoic acid biosynthetic process | 5.84E-05 | 0.022541 |
| Molecular Function | GO:0005310 | dicarboxylic acid transmembrane transporter activity | 0.001102 | 0.022551 |
| Cellular Component | GO:0098862 | cluster of actin-based cell projections | 0.001078 | 0.022809 |
| Molecular Function | GO:0016918 | retinal binding | 0.001235 | 0.024465 |
| Molecular Function | GO:0004952 | dopamine neurotransmitter receptor activity | 0.001414 | 0.027124 |
| Molecular Function | GO:0070011 | peptidase activity, acting on L-amino acid peptides | 0.001514 | 0.028174 |
| Molecular Function | GO:0031406 | carboxylic acid binding | 0.001584 | 0.0286 |
| Cellular Component | GO:0070062 | extracellular exosome | 0.001524 | 0.030707 |
| Biological Process | GO:0016102 | diterpenoid biosynthetic process | 9.27E-05 | 0.031268 |
| Biological Process | GO:0031281 | positive regulation of cyclase activity | 0.000105 | 0.031268 |
| Biological Process | GO:0015711 | organic anion transport | 0.000122 | 0.031268 |
| Biological Process | GO:0051349 | positive regulation of lyase activity | 0.000127 | 0.031268 |
| Biological Process | GO:0031284 | positive regulation of guanylate cyclase activity | 0.000138 | 0.031268 |
| Biological Process | GO:0060267 | positive regulation of respiratory burst | 0.000138 | 0.031268 |
| Biological Process | GO:0015701 | bicarbonate transport | 0.000144 | 0.031268 |
| Cellular Component | GO:1903561 | extracellular vesicle | 0.001841 | 0.033031 |
| Cellular Component | GO:0031362 | anchored component of external side of plasma membrane | 0.001873 | 0.033031 |
| Cellular Component | GO:0043230 | extracellular organelle | 0.001874 | 0.033031 |
| Molecular Function | GO:0004064 | arylesterase activity | 0.002104 | 0.034909 |
| Molecular Function | GO:0010853 | cyclase activator activity | 0.002104 | 0.034909 |
| Molecular Function | GO:0030250 | guanylate cyclase activator activity | 0.002104 | 0.034909 |
| Molecular Function | GO:0008233 | peptidase activity | 0.002215 | 0.035185 |
| Molecular Function | GO:0043177 | organic acid binding | 0.002263 | 0.035185 |
| Molecular Function | GO:0019842 | vitamin binding | 0.002316 | 0.035185 |
| Molecular Function | GO:0008376 | acetylgalactosaminyltransferase activity | 0.002447 | 0.035185 |
| Molecular Function | GO:0022839 | ion gated channel activity | 0.002462 | 0.035185 |
| Molecular Function | GO:0005539 | glycosaminoglycan binding | 0.002464 | 0.035185 |
| Biological Process | GO:0042476 | odontogenesis | 0.000181 | 0.035493 |
| Biological Process | GO:0010038 | response to metal ion | 0.000203 | 0.035493 |
| Biological Process | GO:0030855 | epithelial cell differentiation | 0.000221 | 0.035493 |
| Biological Process | GO:0009887 | animal organ morphogenesis | 0.000228 | 0.035493 |
| Biological Process | GO:0055123 | digestive system development | 0.000247 | 0.035493 |
| Biological Process | GO:0031279 | regulation of cyclase activity | 0.000247 | 0.035493 |
| Biological Process | GO:0010842 | retina layer formation | 0.00025 | 0.035493 |
| Biological Process | GO:0042445 | hormone metabolic process | 0.000261 | 0.035493 |
| Biological Process | GO:0010243 | response to organonitrogen compound | 0.000264 | 0.035493 |
| Biological Process | GO:0070857 | regulation of bile acid biosynthetic process | 0.000266 | 0.035493 |
| Biological Process | GO:0014075 | response to amine | 0.000302 | 0.037405 |
| Biological Process | GO:0051339 | regulation of lyase activity | 0.000302 | 0.037405 |
| Biological Process | GO:0046942 | carboxylic acid transport | 0.000315 | 0.03742 |
| Biological Process | GO:0015849 | organic acid transport | 0.000323 | 0.03742 |
| Biological Process | GO:0046717 | acid secretion | 0.000335 | 0.037524 |
| Biological Process | GO:0031282 | regulation of guanylate cyclase activity | 0.000351 | 0.038109 |
| Molecular Function | GO:0016714 | oxidoreductase activity, acting on paired donors, with incorporation or reduction of molecular oxygen, reduced pteridine as one donor, and incorporation of one atom of oxygen | 0.002922 | 0.039865 |
| Molecular Function | GO:0030249 | guanylate cyclase regulator activity | 0.002922 | 0.039865 |
| Molecular Function | GO:0022836 | gated channel activity | 0.00302 | 0.040307 |
| Biological Process | GO:0065008 | regulation of biological quality | 0.000425 | 0.041421 |
| Biological Process | GO:0042904 | 9-cis-retinoic acid biosynthetic process | 0.000431 | 0.041421 |
| Biological Process | GO:0042905 | 9-cis-retinoic acid metabolic process | 0.000431 | 0.041421 |
| Biological Process | GO:0007494 | midgut development | 0.000453 | 0.041421 |
| Biological Process | GO:0071073 | positive regulation of phospholipid biosynthetic process | 0.000453 | 0.041421 |
| Biological Process | GO:1901698 | response to nitrogen compound | 0.000453 | 0.041421 |
| Cellular Component | GO:0005903 | brush border | 0.002471 | 0.041805 |
| Biological Process | GO:0007423 | sensory organ development | 0.000516 | 0.045901 |
| Biological Process | GO:0030001 | metal ion transport | 0.000546 | 0.046094 |
| Biological Process | GO:0016114 | terpenoid biosynthetic process | 0.000571 | 0.046094 |
| Biological Process | GO:0019755 | one-carbon compound transport | 0.000571 | 0.046094 |
| Biological Process | GO:1904251 | regulation of bile acid metabolic process | 0.000571 | 0.046094 |
| Biological Process | GO:0043010 | camera-type eye development | 0.00059 | 0.046243 |
| Biological Process | GO:0048593 | camera-type eye morphogenesis | 0.000602 | 0.046243 |
| Biological Process | GO:0010043 | response to zinc ion | 0.000613 | 0.046243 |
| Biological Process | GO:0042573 | retinoic acid metabolic process | 0.00064 | 0.047275 |
| Biological Process | GO:0001654 | eye development | 0.00067 | 0.048471 |

Table S3. Results of KEGG enrichment analysis.

| **Pathway ID** | **Pathway** | **out (107)** | **All (8312)** | **Pvalue** |
| --- | --- | --- | --- | --- |
| ko00910 | Nitrogen metabolism | 4 | 17 | 5.43E-05 |
| ko04972 | Pancreatic secretion | 6 | 105 | 0.00227 |
| ko04976 | Bile secretion | 5 | 74 | 0.002563 |
| ko05226 | Gastric cancer | 7 | 153 | 0.003501 |
| ko04964 | Proximal tubule bicarbonate reclamation | 3 | 27 | 0.004846 |
| ko04080 | Neuroactive ligand-receptor interaction | 11 | 352 | 0.005463 |
| ko04310 | Wnt signaling pathway | 7 | 167 | 0.00565 |
| ko04020 | Calcium signaling pathway | 9 | 273 | 0.008427 |
| ko00830 | Retinol metabolism | 4 | 68 | 0.01125 |
| ko04550 | Signaling pathways regulating pluripotency of stem cells | 6 | 150 | 0.012693 |
| ko01230 | Biosynthesis of amino acids | 4 | 79 | 0.018651 |
| ko05030 | Cocaine addiction | 3 | 49 | 0.024904 |
| ko04970 | Salivary secretion | 4 | 93 | 0.031655 |
| ko00220 | Arginine biosynthesis | 2 | 24 | 0.037693 |
| ko05217 | Basal cell carcinoma | 3 | 64 | 0.049101 |
| ko00053 | Ascorbate and aldarate metabolism | 2 | 28 | 0.049945 |
| ko00790 | Folate biosynthesis | 2 | 28 | 0.049945 |

Table S4. Results of GSEA enrichment analysis.

| **ID** | **setSize** | **NES** | **FDR** |
| --- | --- | --- | --- |
| KEGG_WNT_SIGNALING_PATHWAY | 150 | 1.32 | 2.25E-01 |
| KEGG_VASCULAR_SMOOTH_MUSCLE_CONTRACTION | 113 | 1.40 | 1.79E-01 |
| KEGG_TYPE_I_DIABETES_MELLITUS | 41 | -1.61 | 2.55E-01 |
| KEGG_STEROID_HORMONE_BIOSYNTHESIS | 55 | -1.52 | 2.69E-01 |
| KEGG_STARCH_AND_SUCROSE_METABOLISM | 52 | -1.71 | 1.96E-01 |
| KEGG_SPLICEOSOME | 126 | 1.44 | 1.77E-01 |
| KEGG_RNA_POLYMERASE | 29 | 1.47 | 2.45E-01 |
| KEGG_RIBOSOME | 88 | 1.78 | 1.10E-01 |
| KEGG_RETINOL_METABOLISM | 64 | -1.90 | 1.79E-01 |
| KEGG_PRIMARY_IMMUNODEFICIENCY | 35 | -1.49 | 2.45E-01 |
| KEGG_PORPHYRIN_AND_CHLOROPHYLL_METABOLISM | 40 | -1.58 | 2.55E-01 |
| KEGG_O_GLYCAN_BIOSYNTHESIS | 29 | -1.85 | 1.77E-01 |
| KEGG_NITROGEN_METABOLISM | 23 | -1.91 | 1.77E-01 |
| KEGG_NATURAL_KILLER_CELL_MEDIATED_CYTOTOXICITY | 132 | -1.59 | 2.15E-01 |
| KEGG_METABOLISM_OF_XENOBIOTICS_BY_CYTOCHROME_P450 | 69 | -1.49 | 2.28E-01 |
| KEGG_LEISHMANIA_INFECTION | 70 | -1.45 | 2.28E-01 |
| KEGG_JAK_STAT_SIGNALING_PATHWAY | 155 | -1.31 | 2.28E-01 |
| KEGG_INTESTINAL_IMMUNE_NETWORK_FOR_IGA_PRODUCTION | 46 | -2.36 | 1.79E-01 |
| KEGG_HYPERTROPHIC_CARDIOMYOPATHY_HCM | 83 | 1.38 | 2.40E-01 |
| KEGG_HEMATOPOIETIC_CELL_LINEAGE | 85 | -2.16 | 1.79E-01 |
| KEGG_GRAFT_VERSUS_HOST_DISEASE | 37 | -2.08 | 1.79E-01 |
| KEGG_GLYCOSPHINGOLIPID_BIOSYNTHESIS_LACTO_AND_NEOLACTO_SERIES | 26 | -1.75 | 1.77E-01 |
| KEGG_FRUCTOSE_AND_MANNOSE_METABOLISM | 33 | -1.50 | 2.71E-01 |
| KEGG_DRUG_METABOLISM_CYTOCHROME_P450 | 71 | -1.57 | 2.28E-01 |
| KEGG_DILATED_CARDIOMYOPATHY | 90 | 1.49 | 1.77E-01 |
| KEGG_CHEMOKINE_SIGNALING_PATHWAY | 185 | -1.59 | 2.53E-01 |
| KEGG_CARDIAC_MUSCLE_CONTRACTION | 74 | 1.43 | 1.93E-01 |
| KEGG_AUTOIMMUNE_THYROID_DISEASE | 50 | -1.92 | 1.79E-01 |
| KEGG_ASCORBATE_AND_ALDARATE_METABOLISM | 25 | -1.66 | 1.79E-01 |
| KEGG_ALLOGRAFT_REJECTION | 35 | -1.81 | 1.83E-01 |
